# Role of the *ALK* gene and PI3K/Akt/NF-κB signaling pathway in cervical cancer precancerous lesions

**DOI:** 10.3389/fonc.2025.1619703

**Published:** 2025-09-04

**Authors:** Zhengran Sun, Ding Qi, Li Liu, Wenxia Ai, Buwei Han, Shimeng Wang, Mingge Liang, Yonggang Xia

**Affiliations:** ^1^ Harbin Medical University, Harbin, Heilongjiang, China; ^2^ Heilongjiang Provincial Traditional Chinese Medicine Hospital, Harbin, Heilongjiang, China; ^3^ Heilongjiang University Of Chinese Medicine, Harbin, Heilongjiang, China; ^4^ The 2nd Affiliated Hospital of Heilongjiang University of Traditional Chinese Medicine, Harbin, Heilongjiang, China; ^5^ The 1st Affiliated Hospital of Heilongjiang University of Traditional Chinese Medicine, Harbin, Heilongjiang, China; ^6^ Harbin University of Commerce, Harbin, Heilongjiang, China

**Keywords:** cervical precancerous lesions, ALK, PI3K/Akt/NF-κB, prognosis, diagnosis

## Abstract

**Introduction:**

This study aimed to elucidate the molecular mechanisms underlying cervical precancerous lesions by employing bioinformatic approaches to identify key genes and signaling pathways.

**Methods:**

A comprehensive strategy was adopted, beginning with the analysis of GEO datasets to determine differentially expressed genes (DEGs) between cervical squamous intraepithelial lesions (CSILs) and normal cervical tissues. Protein–protein interaction (PPI) networks were constructed using STRING 11.0 and visualized with Cytoscape 3.7.2. Functional annotation through Gene Ontology (GO) and KEGG pathway enrichment using DAVID revealed biological processes, cellular components, molecular functions, and signaling pathways associated with the DEGs. Gene Set Enrichment Analysis (GSEA) further refined critical genes and enriched pathways. Similarly, quantitative real-time PCR (qRT-PCR) was performed on cervical biopsy samples from eligible patients to validate the bioinformatic predictions.

**Results:**

The analysis identified 371 common DEGs across datasets, uncovering 102 biological processes, 33 cellular components, 15 molecular functions, 29 significantly enriched pathways, and three key genes. Clinical correlations demonstrated that lesion severity was associated with age, vaginal microbiota composition, and activation of the ALK gene and PI3K/AKT/NF-κB signaling axis. qRT-PCR confirmed increased *ALK* expression and PI3K/AKT/NF-κB pathway activity in high-grade lesions, supporting their involvement in CSIL pathogenesis. These findings highlight the potential of this research to guide the development of targeted therapies and personalized treatment strategies for cervical precancerous lesions.

**Discussion:**

By pinpointing the molecular drivers of disease, this work provides a foundation for interventions aimed at precisely modulating these pathways, improving clinical outcomes and reducing the overall burden of cervical cancer.

## 1 Introduction

Cervical precancerous lesions, clinically referred to as cervical intraepithelial neoplasia (CIN), are strongly associated with the development of invasive cervical cancer. Based on pathological grading, CIN is classified into CIN I, CIN II, and CIN III. These lesions represent a pivotal stage in cervical carcinogenesis, and their further categorization into low-grade squamous intraepithelial lesions (LSIL) and high-grade squamous intraepithelial lesions (HSIL), often guided by biomarkers such as p16 expression, provides important insights into disease progression (1). The transition from normal cervical epithelium to precancerous lesions and subsequently to invasive cancer is a complex, multistep process characterized by dysregulation of numerous genes and signaling pathways (2). Although persistent infection with high-risk human papillomavirus (HPV) is recognized as the primary etiological factor for cervical cancer (3), it is noteworthy that only a subset of HPV-infected individuals progress to malignancy. This observation underscores the role of additional genetic susceptibilities and environmental influences in driving the advancement of cervical precancerous lesions toward invasive cancer (4).

In recent years, the investigation of gene–disease relationships has gained considerable importance in elucidating disease pathogenesis (5). In the context of cervical cancer, numerous studies have identified specific genes and signaling pathways that contribute to disease development and progression. For instance, the *PTEN* gene, a critical tumor suppressor, is abundantly expressed in normal cervical tissue, where it maintains the balance between cell proliferation and apoptosis (6). However, *PTEN* expression is frequently downregulated or absent in precancerous lesions and cervical cancer tissues (7), diminishing its inhibitory effect on the PI3K/Akt pathway and facilitating abnormal cell proliferation and tumorigenesis (8). Similarly, the TGF-β signaling pathway plays a pivotal role in tumor initiation and progression, with TGFBR2 serving as one of its core components. Loss or reduced expression of TGFBR2 has been implicated in promoting tumor development and progression (9). Another gene of interest, *eEF1A2*, is associated with the onset and progression of various malignancies, including ovarian (10), liver cancer (11), and prostate cancer (12). While direct evidence for its role in cervical cancer is limited, previous findings suggest that eEF1A2 may contribute to cervical carcinogenesis by inhibiting apoptosis and increasing cancer cell proliferation (13). Beyond individual genes, dysregulation of signaling pathways also influences disease susceptibility. The cGAS–STING signaling pathway, a critical mediator of immune responses to viral infections, has been shown to play a role in cervical disease, with abnormal activation potentially linked to increased susceptibility to cervical precancerous lesions (14). Current evidence indicates that the molecular mechanisms driving the development of HSIL are multifaceted, involving diverse biological processes and interconnected signaling pathways (15). However, genetic studies addressing HSIL-specific molecular features remain relatively scarce.

Therefore, this study seeks to leverage bioinformatics tools and high-throughput sequencing data to identify genes and pathways closely associated with HSIL, aiming to discover more specific and sensitive molecular indicators. These findings may enhance genetic research on HSIL and support the development of reliable early diagnostic biomarkers, ultimately improving early detection, enabling timely intervention and treatment, and reducing the incidence and mortality of cervical cancer.

Anaplastic lymphoma kinase (ALK) is a receptor tyrosine kinase encoded by the *ALK* gene located on the short arm of chromosome 2. It plays a critical role in cellular signaling pathways that regulate proliferation, maintaining normal cellular homeostasis through the control of cell growth and division (8). The phosphatidylinositol-3-kinase (PI3K)/Akt/NF-κB signaling cascade, in conjunction with ALK, is deeply involved in cellular proliferation, survival, migration, and inflammatory responses, positioning these pathways as potential focal points for understanding the pathogenesis of CSIL in the setting of human papillomavirus (HPV) infection. Genetic alterations of *ALK*, including recombination, fusion, mutation, amplification, and alternative splicing, have been identified in multiple malignancies. Moreover, *ALK* has been shown to mediate NF-κB signaling, influencing the activation of inflammasomes in macrophages and modulating inflammatory responses.

This study represents an innovative investigation into the key genes and signaling networks implicated in CSIL, proposing for the first time a pathogenic mechanism in which ALK-mediated activation of the PI3K/Akt/NF-κB pathway contributes to lesion development. Future research should aim to clarify the precise molecular interactions between these pathways and HPV infection, while also exploring targeted therapeutic strategies that modulate these pathways to prevent or manage CSIL and reduce the progression to cervical cancer.

## 2 Materials and methods

### 2.1 Patient collection

This study was designed as a case–control investigation. Patients diagnosed with SIL and admitted to the First Affiliated Hospital of Heilongjiang University of Traditional Chinese Medicine between March 2023 and February 2024, who met the established diagnostic criteria, were enrolled.

#### 2.1.1 Diagnostic criteria

The LSIL were defined as lesions confined to the lower one-third of the cervical epithelium. Cytological features included mild disturbance of nuclear polarity, minimal mitotic activity, evenly distributed chromatin, and the presence of koilocytes characteristic of HPV infection. The HSIL involved two-thirds or more of the cervical epithelium. Cells showed significant loss of nuclear polarity, an increased nuclear-to-cytoplasmic ratio, and extension of atypical cells to the upper two-thirds or full thickness of the epithelium.

#### 2.1.2 Inclusion criteria

Eligible participants were women aged 20–50 years with a history of sexual activity, whose cervical histopathology met the diagnostic criteria for LSIL or HSIL, and who had not received vaginal or cervical medications in the previous month. Patients in the lesion group were required to have a confirmed diagnosis from the hospital’s pathology department. Control group participants were required to have negative HPV and ThinPrep cytology test (TCT) results. All participants were fully informed of the study objectives and procedures and provided written informed consent.

#### 2.1.3 Exclusion criteria

Exclusion criteria included: (1) pathological diagnoses from external institutions not reviewed or confirmed by the hospital, or cases with diagnostic discrepancies; (2) before treatment with loop electrosurgical excision procedure (LEEP) or conization for cervical lesions; (3) detection of abnormal glandular cells or histologically confirmed malignant lesions; (4) coexisting severe systemic illness requiring urgent intervention or advanced cardiac, hepatic, or renal dysfunction; (5) irregular vaginal bleeding during pregnancy, lactation, menstruation, or prolonged abnormal bleeding; and (6) poor compliance, incomplete clinical data, or withholding of relevant medical information.

This study was reviewed and approved by the Ethics Committee of the First Affiliated Hospital of Heilongjiang University of Traditional Chinese Medicine (Approval No. HZYLLKY202300701). All procedures were conducted following the relevant ethical guidelines and regulations. Written informed consent was obtained from all participants before enrollment.

### 2.2 Data collection

The Gene Expression Omnibus (GEO) database (https://www.ncbi.nlm.nih.gov/geo/) is a publicly accessible repository containing gene expression profiles for a wide range of diseases. For this study, we retrieved human cervical disease gene expression datasets that included samples from normal cervical tissue, LSIL, HSIL, and cervical cancer (CC). Comprehensive analyses were performed on the eligible datasets to identify key genes consistently expressed across different datasets.

Dataset selection criteria were as follows: (1) inclusion of tissue samples from HSIL or SIL; (2) availability of complete technical details and platform information relevant for analysis; and (3) inclusion of normal cervical tissue samples as controls.

### 2.3 Identify differentially expressed genes

Differentially expressed genes (DEGs) were identified using the GEO2R tool (https://www.ncbi.nlm.nih.gov/geo/geo2r/) within the GEO database. Data processing was performed with the limma R package (version 4.2.1). Genes meeting the criteria of *P<* 0.05 and log fold change (LogFC) > 2.0, LogFC< –2.0, or |Log2FC| > 1 were classified as DEGs.

To determine key genes consistently expressed across datasets, Venn diagrams were generated to analyze overlapping DEGs among the selected datasets. The shared DEGs identified through this overlap were extracted for further analysis.

### 2.4 Construction of protein-protein interaction network

The target genes were uploaded to the UniProt database (https://www.uniprot.org) to obtain standardized gene names. The converted gene information was then submitted to the STRING database (https://cn.string-db.org/cgi/input.pl) for PPI analysis, with the organism set to Homo sapiens and all other parameters maintained at default settings. PPI network visualization was performed using Cytoscape software (version 3.9.1), and the CytoHubba plugin was applied to identify hub genes within the network. These hub genes were subsequently selected as candidate DEGs for further analysis.

### 2.5 Gene ontology and kyoto encyclopedia of genes and genomes pathway analysis

The DAVID online platform (https://david.ncifcrf.gov) was used to perform functional annotation and enrichment analysis of the differentially expressed genes, with the organism set to *Homo sapiens* and the gene identifier specified as “official gene symbol.” A threshold of *P<* 0.05 was considered statistically significant. Visualization of the DAVID analysis results was performed using the online bioinformatics tool Weishengxin (http://www.bioinformatics.com).

### 2.6 Gene set enrichment analysis predicts key genes

The gene symbols were uploaded to the Weishengxin platform, where the “Hallmark gene sets” were selected for gene set enrichment analysis. This process enabled the prediction and screening of key genes and potential targets. Simultaneously, enrichment analysis was performed to identify the most relevant gene modules associated with the disease, elucidating the critical biological processes involved in its pathogenesis.

### 2.7 qRT-PCR

A total of 70 mg of tissue was collected from each cervical specimen that met the inclusion and exclusion criteria, including normal, LSIL, and HSIL samples. Total RNA was extracted by adding 1 mL of RNAkey™ Reagent (Beijing Saiwen Innovation Biotechnology Co., Ltd., China). RNA purity and concentration for each sample were measured before reverse transcription.

Quantitative real-time PCR (qRT-PCR) was performed using the SYBR Premix Ex Taq II kit (TaKaRa Biotechnology, Japan) on the ABI QuantStudio 12K Flex Real-Time PCR System (Foster City, CA, USA). The PCR reaction mixture consisted of DNA template, primers, and 2× SYBR Green qPCR Master Mix. Reaction mixtures were prepared according to **Table 1**, with a total volume of 20 µL per well and three technical replicates per sample. The average of the triplicate values was used for analysis. Primer sequences for each target are listed in **Table 2**.

**Table 1:** Reaction system configuration table.

Reagent components	Volume
2×SYBR Green qPCR MasterMix II	10µL
Forward primer, 10µM	1µL
Reverse primer, 10µM	1µL
Template DNA	1.5µL
Nuclease-FreeWater	6.5µL

**Table 2:** Primer sequence design.

Primer name	Sequence:5′- 3′
STK33	F: GAAAAGTTTCTCCCGGTGCAG
	R: TTTATCTGGCTCCCCATCGC
RPS14	F:AGCTTGTGAAAAATGGCACCTC
	R:TTCATCCCACCAGTCACAC
ALK	F:CCAGACTAACATGACTCTGCC
	R: AGCCTCCCTGGATCTCCATA
PIK3CA	F:GGACCCGATGCGGTTAGAG
	R:ATCAAGTGGATGCCCCACAG
AKT1	F:GGACAAGGACGGGCACATTA
	R: CGACCGCACATCATCTCGTA
NF-κB	F:AATGGGCTACACCGAAGCAA
	R:CTGTCGCAGACACTGTCACT
IκB-α	F:AAGTGATCCGCCAGGTGAAG
	R:CTGCTCACAGGCAAGGTGTA
GAPDH	F:CTCGCTCCTGGAAGATGGTG
	R:GCAAAGTAGAAAAGGGCAAC

### 2.8 Statistical analyses

For cell-based experiments, data represent the results of three independent replicates. Unless otherwise specified, all values are expressed as mean ± standard deviation (SD). Statistical analyses were conducted using SPSS software (version 25.0). One-way ANOVA was applied for datasets meeting the assumption of normality, while the rank-sum test was used for non-normally distributed data. GraphPad Prism software (version 9.5.1) was used for data visualization. A *P<* 0.05 was considered statistically significant, and a *P<* 0.01 was regarded as highly significant.

## 3 Results

### 3.1 Bioinformatics analysis results

#### 3.1.1 Determination of the dataset

A search of the GEO database using the terms “cervical intraepithelial neoplasia,” “cervical HSIL,” and “cervical high-grade intraepithelial lesion” initially identified 588 eligible datasets. Following detailed screening, inspection, and data correction, two datasets, GSE63514 and GSE75132, were selected for comparative analysis. Both datasets were generated using high-throughput sequencing on the GPL570 platform, which has a detectable nucleotide (nt) length of 600. The Affymetrix Human Genome U133 Plus 2.0 Array was employed, providing comprehensive coverage of the U133 set of the human genome along with 6,500 additional genes and over 47,000 transcripts, ensuring high sensitivity and accuracy of detection (16).

The GSE63514 dataset comprises 128 sequencing samples, including tissue samples from 24 normal controls, 14 cases of CIN I, 22 cases of CIN II, 40 cases of CIN III, and 28 cases of cervical cancer. The GSE75132 dataset contains 41 samples, including 11 normal controls, 10 cases of persistent HPV16 infection, 4 cases of CIN II, 9 cases of CIN III, and 7 cases of cervical cancer.

#### 3.1.2 GEO 2R processing results

**Figure 1:**
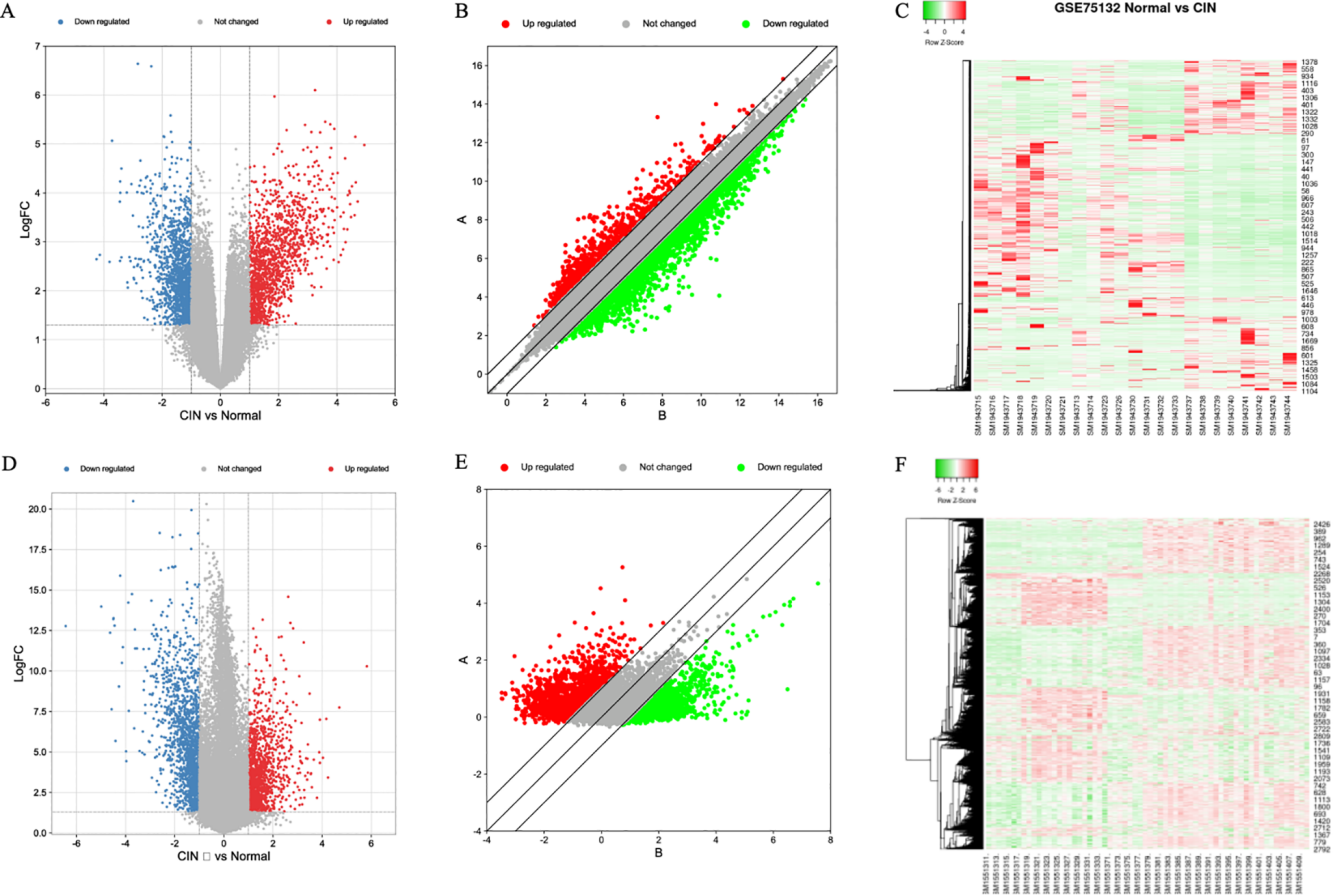
Gene expression in two datasets. **(A)** GSE75132 Volcano Map, **(B)** GSE75132 Scatter plot, **(C)** GSE75132 heat map, **(D)** GSE63514 Volcano Map, **(E)** GSE63514 scatter plot, **(F)** GSE63514 Heat Map.

The basic characteristics of the two datasets were analyzed as follows;

In the GSE75132 dataset, baseline alignment was first performed, followed by comparative analysis. A volcano plot (**Figure 1A**) and scatter plot (**Figure 1B**) were generated to visualize the differential gene expression. In **Figure 1A**, blue indicates downregulated genes and red indicates upregulated genes. In **Figure 1B**, green denotes downregulated genes, while red denotes upregulated genes. A total of 2,266 differentially expressed genes (DEGs) were identified, including 951 significantly upregulated genes and 1,315 significantly downregulated genes (**Figure 1C**). In **Figure 1C**, green represents downregulated genes, while red represents upregulated genes.

In the GSE63514 dataset, analysis of normal tissues versus high-grade lesion tissues revealed 2,208 DEGs (**Figures 1D, E**). Among these, 1,203 genes were significantly downregulated and 1,005 genes were significantly upregulated (**Figure 1F**). In **Figure 1D**, blue represents downregulated genes and red represents upregulated genes; in **Figure 1E**, green denotes downregulated genes and red denotes upregulated genes; in **Figure 1F**, green indicates downregulated genes and red indicates upregulated genes.

#### 3.1.3 Screening of differentially expressed genes

**Figure 2:**
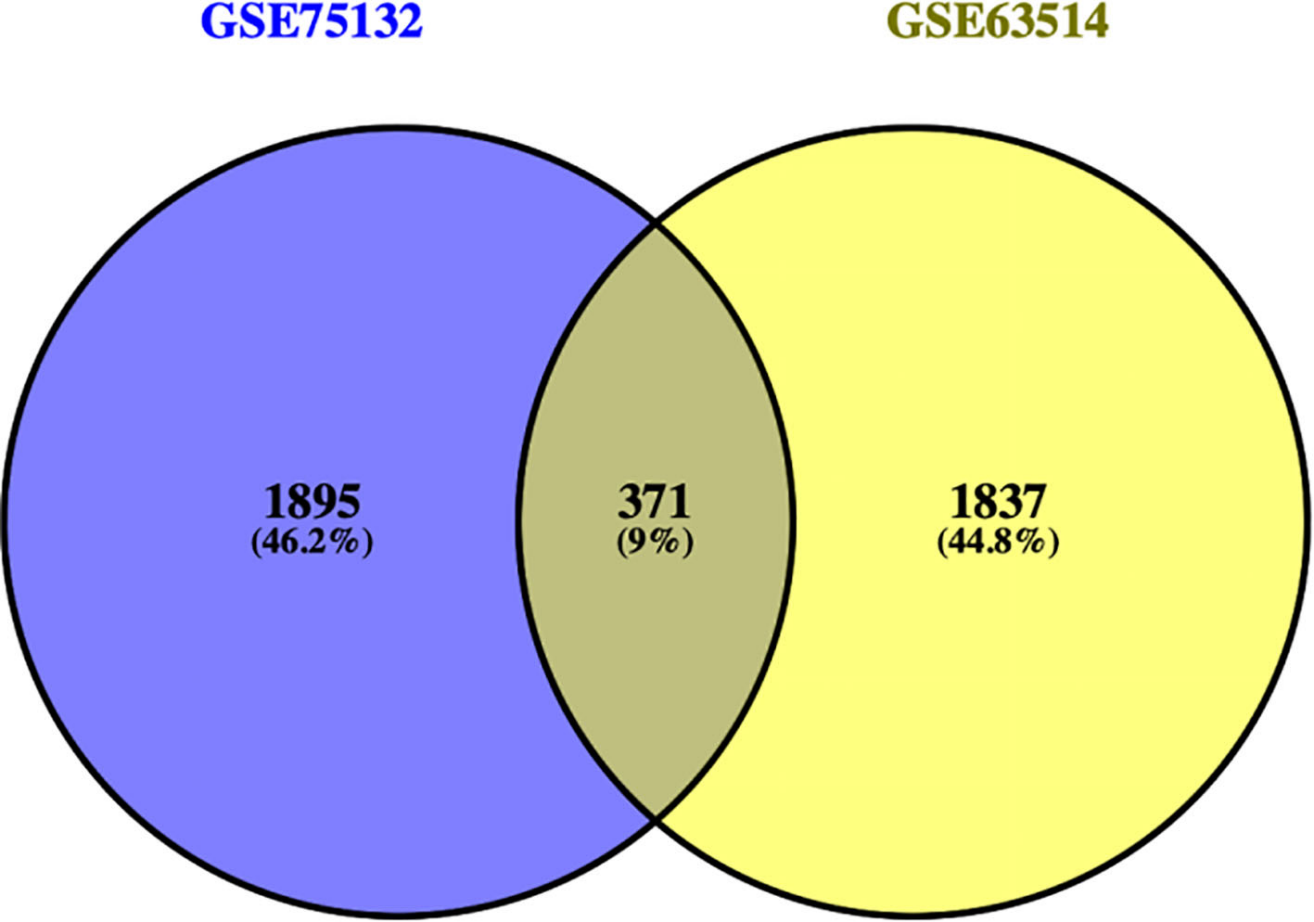
Venny plot.

The DEGs from the two datasets were uploaded separately to the Venny tool (https://bioinfogp.cnb.csic.es/tools/venny/index.html) to generate a Venn diagram (**Figure 2**). The GSE75132 dataset contained 2,266 DEGs, while the GSE63514 dataset contained 2,208 DEGs. A total of 371 DEGs were found to be shared between the two datasets. Further analysis of these 371 common DEGs revealed 221 downregulated genes and 150 upregulated genes.

#### 3.1.4 PPI network analysis results

**Figure 3:**
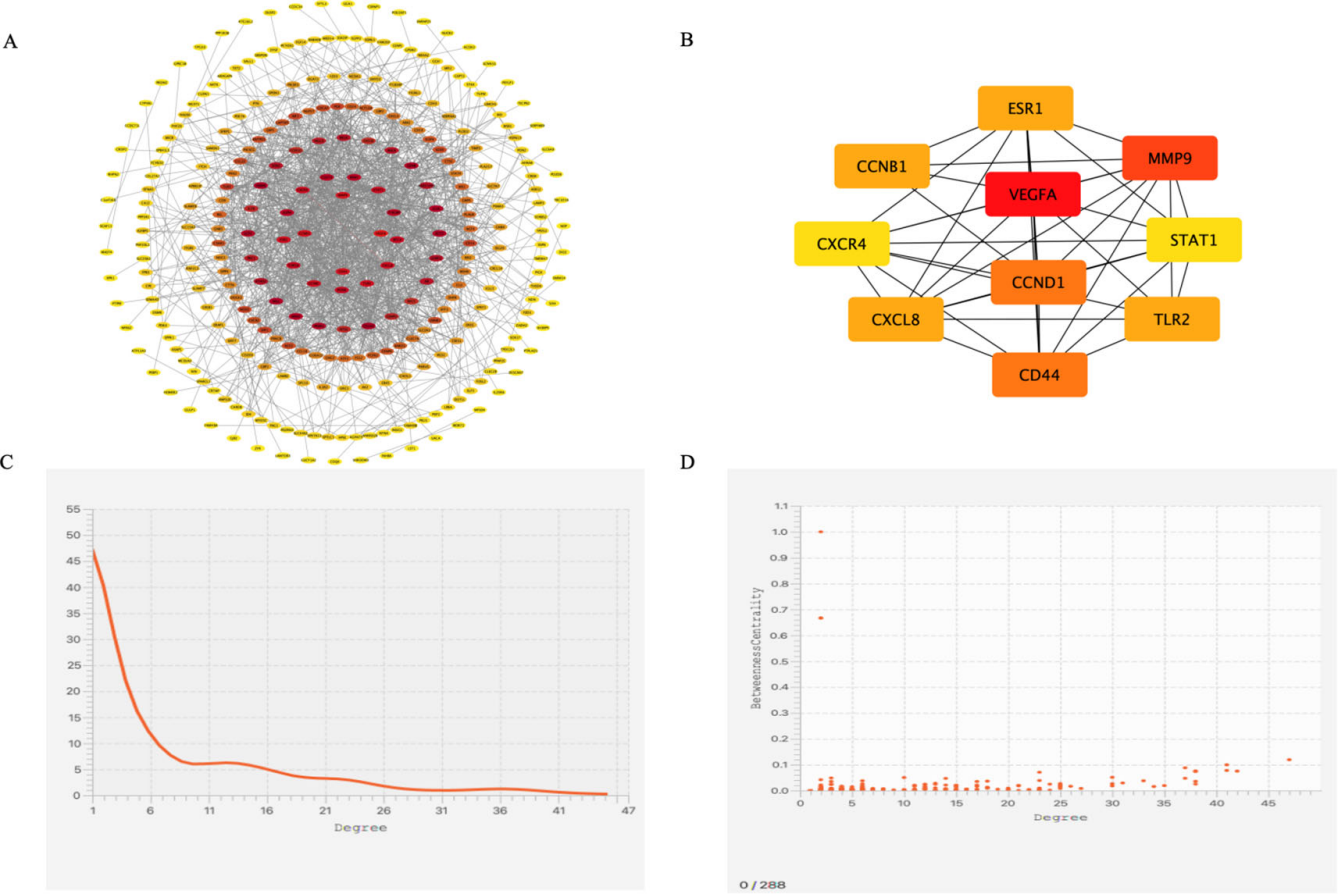
PPI Network and Cytoscape Analysis. **(A)** PPI network diagram of genes, **(B)** The top 10 key genes in terms of degree ranking, **(C)** Degree distribution map of differentially co-expressed genes, **(D)** Degree correlation analysis of differentially co-expressed genes.

The results from the STRING database analysis were downloaded and imported into Cytoscape software to construct a PPI network for the 371 differentially expressed genes. The resulting network comprised 288 nodes and 1,274 edges (**Figure 3A**). Using the CytoHubba plugin, the top 10 hub genes within the PPI network were identified (**Figure 3B**). These included vascular endothelial growth factor A (*VEGFA*), matrix metalloproteinase-9 (*MMP9*), hyaluronan receptor CD44 (*CD44*), cyclin D1 (*CCND1*), cyclin B1 (*CCNB1*), C-X-C motif chemokine ligand 8 (*CXCL8*), estrogen receptor 1 (*ESR1*), Toll-like receptor 2 (*TLR2*), C-X-C chemokine receptor 4 (*CXCR4*), and signal transducer and activator of transcription 1 (*STAT1*). The average degree value of the PPI network is 7.26 (**Figure 3C**), and the correlation between genes is good (**Figure 3D**).

#### 3.1.5 GO analysis

**Figure 4:**
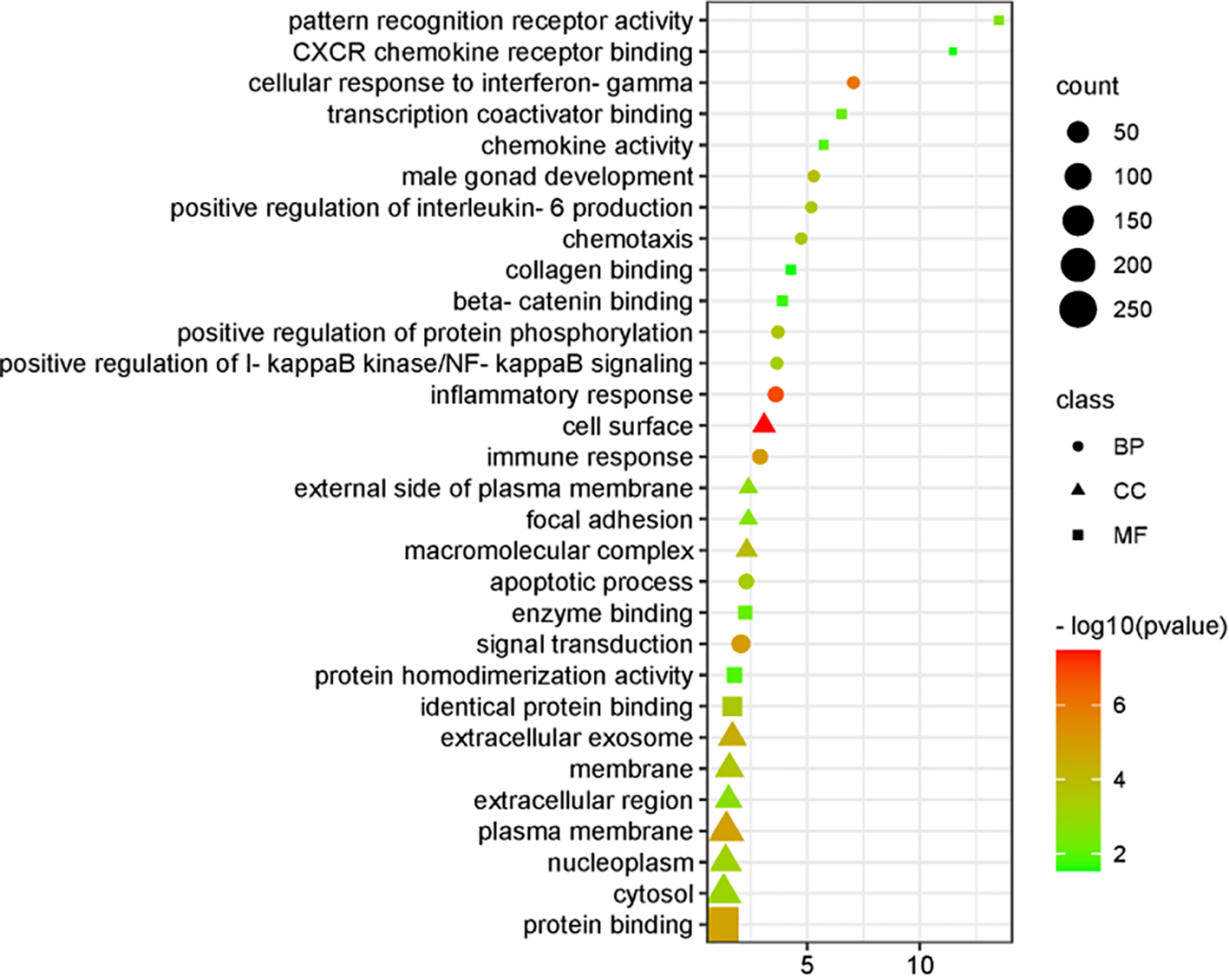
Visual display of GO analysis.

Analysis of the differentially expressed genes identified 102 enriched biological processes (BP), 33 cellular components (CC), and 15 molecular functions (MF). The top 10 terms from each category were selected for GO visualization (**Figure 4**). The results revealed several processes closely associated with CSIL.

Key biological processes included inflammatory response, cellular response to interferon-γ, immune response, signal transduction, positive regulation of protein phosphorylation, positive regulation of interleukin-6 production, apoptotic processes, and positive regulation of IκB kinase/NF-κB signaling. The enriched cellular components were primarily associated with cytoplasmic constituents and the extracellular environment. The dominant molecular functions were related to protein binding, enzyme binding, and interactions with associated cofactors and protein complexes.

#### 3.1.6 KEGG pathway screening

**Figure 5:**
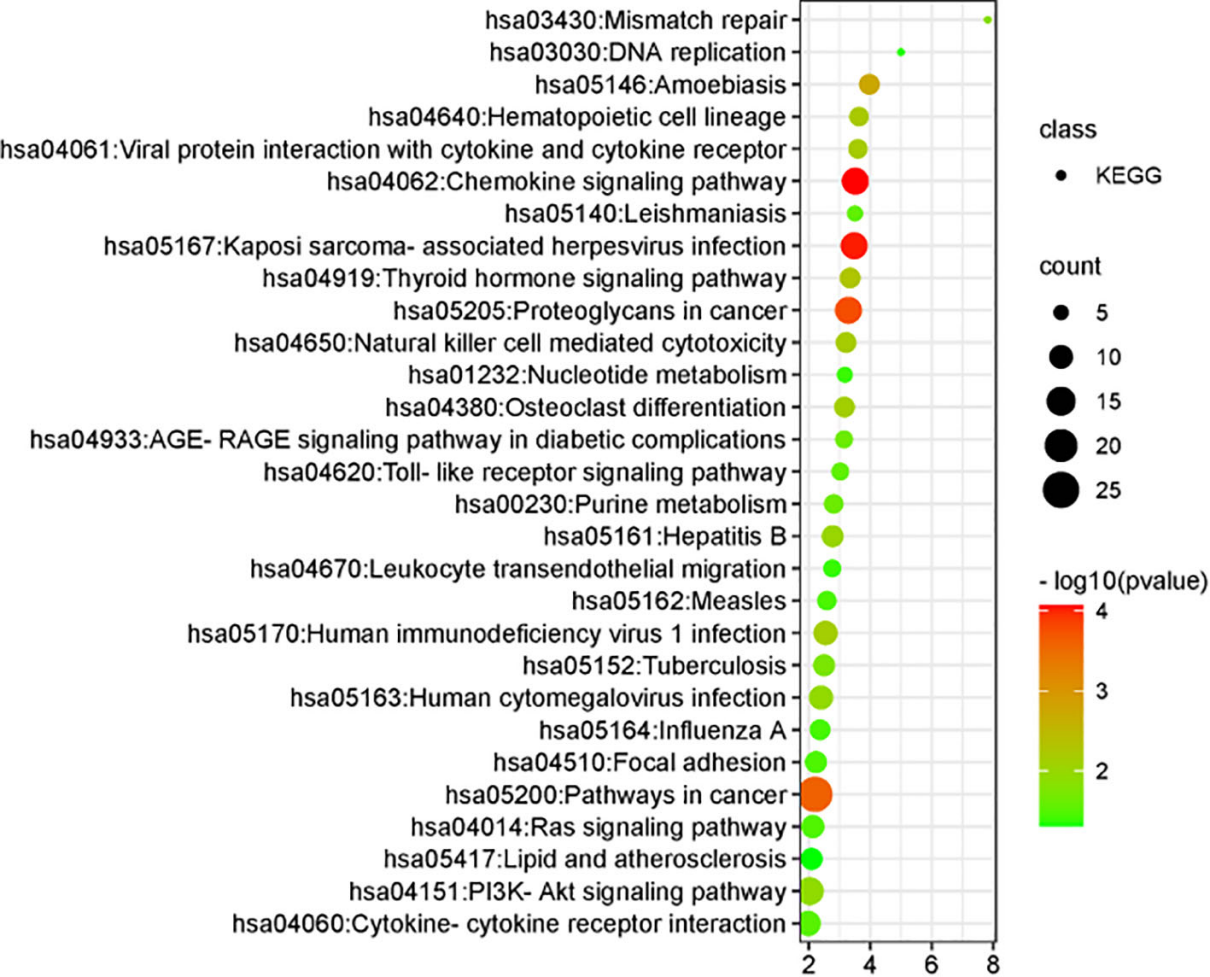
KEGG pathway visualization analysis.

Comparative analysis identified 29 signaling pathways associated with cervical HSIL lesions. The key pathways are illustrated in **Figure 5**. Importantly enriched pathways included Kaposi sarcoma-associated herpesvirus infection, proteoglycans in cancer, general cancer pathways, interactions between viral proteins and cytokines or cytokine receptors, natural killer cell-mediated cytotoxicity, human immunodeficiency virus type 1 infection, human cytomegalovirus infection, the PI3K–Akt signaling pathway, Toll-like receptor signaling pathway, cytokine–cytokine receptor interaction, Ras signaling pathway, nucleotide metabolism, leukocyte transendothelial migration, DNA replication, and lipid and atherosclerosis-related processes.

#### 3.1.7 GSEA key gene screening

**Figure 6:**
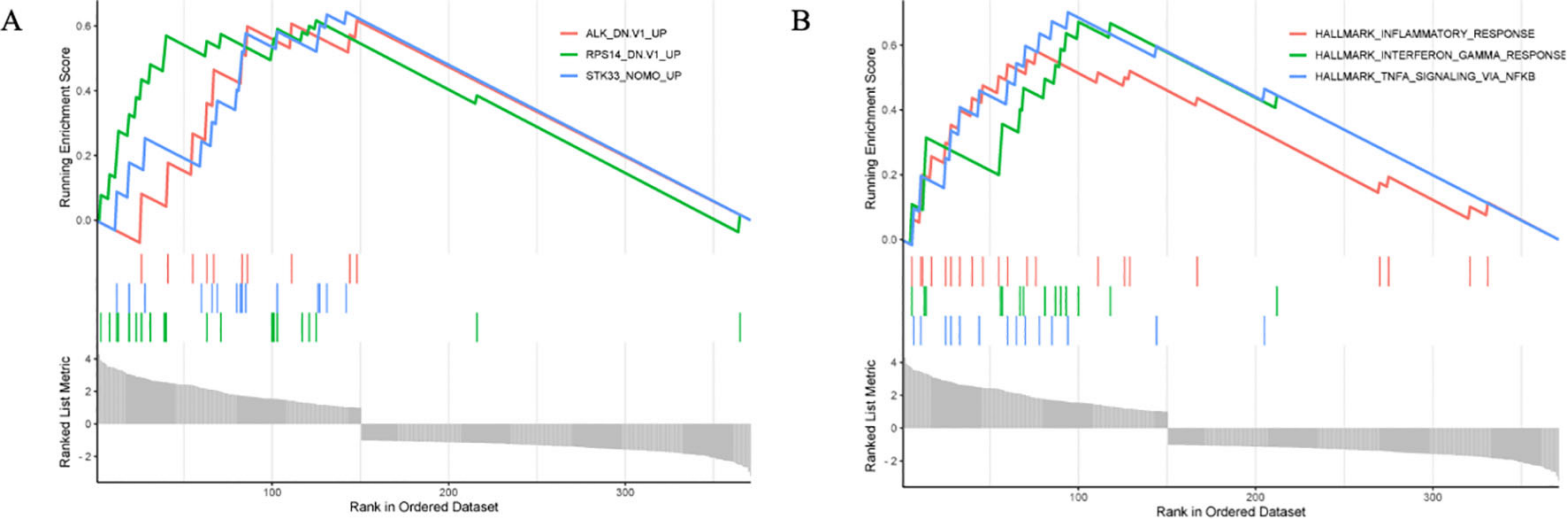
GSEA analysis results. **(A)** Key gene prediction, **(B)** Prediction of Key Gene Enrichment Module.

GSEA enrichment analysis predicted three key genes closely associated with the disease: serine/threonine kinase 33 (*STK33*), ribosomal protein S14 (*RPS14*), and anaplastic lymphoma kinase (*ALK*). The expression levels of all three genes demonstrated an upward trend in the disease samples (**Figure 6A**).

GSEA identified the three most relevant biological processes for this disease: the TNF-α signaling pathway mediated by NF-κB, the interferon-γ response, and the immune response (**Figure 6B**).

Studies have demonstrated that STK33 is expressed across multiple tissues, with the strongest hybridization signals observed in the testes, fetal lungs, and heart, followed by the pituitary gland, kidneys, pancreas, thyroid gland, and uterus. Moderate signals are seen in the aorta, hematopoietic system, and digestive tract, whereas no detectable hybridization is observed in nervous system tissues (17). In tumor-related diseases, STK33 has been shown to suppress mitochondrial apoptosis by inhibiting BAD activity, enhancing tumor cell survival and proliferation. These findings suggest that STK33 may represent a potential therapeutic target for malignancies driven by oncogenic mutations (18).

Literature evidence indicates that RPS14 can disrupt the MDM2–p53 interaction, resulting in significant accumulation of p53. Elevated p53 levels can induce cell cycle arrest, promote DNA damage repair, and trigger cellular senescence and apoptosis (19). RPS14, a component of the 40S ribosomal subunit, is involved in erythroid differentiation and has been implicated in hematological disorders such as myelodysplastic syndrome and 5q syndrome (20). In colon cancer, overexpression of *RPS14* has been reported to activate the PI3K/Akt signaling pathway, enhancing tumor cell survival (21).

Alterations in the *ALK* gene, including recombination, fusion, mutation, amplification, and alternative splicing, have been identified in various malignancies. *ALK* also mediates NF-κB signaling and promotes activation of the NLRP3 inflammasome in macrophages. These properties suggest that ALK-targeted interventions may represent a novel therapeutic strategy for NLRP3 inflammasome–mediated diseases (22, 23).

### 3.2 Data analysis

#### 3.2.1 Basic patient information

A total of 19 normal cervical tissues, 14 LSIL tissues, and 15 HSIL tissues were collected from outpatient and inpatient cases. Normal cervical tissues were obtained from patients undergoing total hysterectomy for non-neoplastic conditions at the First Affiliated Hospital of the Heilongjiang University of Chinese Medicine. All normal tissue donors tested negative for both HPV and TCT. LSIL and HSIL cervical tissues were confirmed by the hospital’s Department of Pathology and met the inclusion and exclusion criteria described earlier.

#### 3.2.2 Comparison of basic age information of patients in different groups

Comparison of age distribution among the groups revealed statistically significant differences (*P<* 0.01). In the control group, most patients were aged 40–50 years and primarily underwent total hysterectomy for benign conditions such as adenomyosis or multiple uterine fibroids. LSIL cases were more common in younger patients, predominantly within the 20–30 year and 30–40 year age groups. However, HSIL cases were mainly concentrated in the 30–40 year and 40–50 year age ranges (**Table 3**).

**Table 3:** Comparison of age distribution among different groups [*n* (%)].

Age	*n*	20-30	>30-40	>40-50
Control	19	0 (0)	0 (0)	19 (100)
LSIL	14	5 (35.71)	6 (42.86)	3 (21.43)
HSIL	15	1 (6.67)	6 (40)	8 (53.33)
χ^2^	25.08			
*P*	0.00**			

*P<0.05, **P<0.01.

This study found that the age of onset for LSIL was primarily concentrated in the 30–40-year age group, reflecting a younger onset pattern. However, HSIL onset was more common in the 40 to 50-year age group. Statistical analysis indicated a positive correlation between age and disease severity, which may be associated with the prolonged duration of viral infection.

The relationship between age and disease severity can be summarized as follows:

*1. Variation in lesion severity across age groups*.

Chang HK et al. reported that the peak onset age for LSIL is 25–29 years, for HSIL is 30–34 years, and for cervical cancer is 70–74 years. Lesion severity tends to increase with age (24). Younger patients generally demonstrate higher rates of lesion regression and complete remission, with lower rates of progression (25).

*2*. *Differences in the natural regression rate of SIL by age*.

HSIL represents a precancerous condition that can progress to cervical cancer. Meta-analysis has shown that the regression rate differs by lesion grade. For CIN 1, spontaneous regression occurs in approximately 40% of cases. Advanced lesions (CIN 2 or CIN 3) have a higher risk of progression, with CIN 2 showing a progression rate of about 10.28%. Age is negatively correlated with regression rate; older patients show lower spontaneous regression rates (26).

*3*. *Association of age with abnormal HPV and TCT results*.

The average latency between carcinogenic HPV infection and the development of cervical cancer is approximately 25–30 years. With advancing age, the prevalence of high-risk HPV (HR-HPV) positivity and abnormal TCT findings increases (27). HPV infection is most prevalent among younger and middle-aged women, particularly those aged 25–35 years, due to higher sexual activity. However, in older women, factors such as lower educational levels, reduced immune function, and hormonal changes contribute to a higher detection rate of SIL (28).

#### 3.2.3 Distribution of other factors in each group of patients

Analysis of baseline characteristics across the patient groups revealed no significant differences (*P* > 0.05) in parity, smoking history, alcohol consumption, vaginal microbiota diversity, or microbiota density between the LSIL, HSIL, and control groups. However, significant differences were observed in the age at first sexual intercourse and the number of sexual partners (*P<* 0.05 and *P<* 0.01, respectively).

Statistical evaluation (**Table 4**) indicated that age, age at first sexual intercourse, and number of sexual partners were associated with the occurrence of SIL. In comparison, parity, smoking history, alcohol consumption, vaginal microbiota diversity, and microbiota density showed no significant association in this study. Comparative analysis further demonstrated that the lesion groups (LSIL and HSIL) were characterized by earlier sexual debut and a higher number of sexual partners compared with the control group. *This study revealed that host factors (including age, vaginal microbiota composition, and immune status) and environmental factors (such as early sexual behavior, multiple sexual partners, and smoking tendency) jointly affect the activation level of the PI3K/Akt/NF - κ B signaling pathway. These findings emphasize the importance of host environment interaction in the development of cervical lesions. In particular, the IFN - γ response shown by GSEA analysis suggests a close relationship between this pathway and host immune response, providing a theoretical basis for the development of combination therapy strategies that combine immune regulation (such as enhancing antiviral response) and targeted pathway inhibition (such as using ALK or PI3K inhibitors), which may improve the therapeutic effect of cervical precancerous lesions.*

**Table 4:** Distribution of other factors in each group [*n* (%)].

Group	*n*	Gravidity and parity history		Smoke		Insobriety		Age of first sexual intercourse		Number of sexual partners		Diversity of vaginal microbiota		Vaginal microbiota density	
		≤2	>2	No	Yes	No	Yes	≤20	>20	<2	≥2	+, ++++	++, +++	+, ++++	++, +++
Control	19	17 (89.47)	2 (10.53)	19 (100)	0 (0)	18 (94.74)	1 (5.26)	6 (31.58)	13 (68.42)	17 (89.47)	2 (10.53)	18 (94.74)	1 (5.26)	18 (94.74)	1 (5.26)
LSIL	14	13 (92.86)	1 (7.14)	14 (100)	0 (0)	9 (64.29)	5 (35.71)	11 (78.57)	3 (21.43)	3 (21.43)	11 (78.57)	1 (7.14)	13 (92.86)	2 (14.29)	12 (85.71)
HSIL	15	12 (80)	3 (20)	14 (93.33)	1 (6.67)	13 (86.67)	2 (13.33)	11 (73.33)	4 (26.67)	2 (13.33)	13 (86.67)	2 (13.33)	13 (86.67)	2 (13.33)	13 (86.67)
χ^2^		1.17		2.02		4.95		8.98		25.19		0.93		1.14	
*P*		0.64		0.60		0.07		0.01*		0.00**		0.82		0.60	

*Through comparative analysis, it was found that there were no differences among the three groups of patients in terms of parity, smoking, alcohol consumption, microbial diversity, and microbial density (*P*>0.05), but there were differences in age at first sexual intercourse and number of sexual partners(*P<0.05, **P<0.01).

#### 3.2.4 HPV and TCT test results

Analysis of HPV genotyping and TCT examination results in the LSIL and HSIL groups revealed that, compared with the LSIL group, the HSIL group had a significantly higher detection rate of high-risk HPV types 16 and 18 (*P<* 0.05). The LSIL group, however, showed relatively higher detection rates of other high-risk HPV types.

In terms of cytology, most patients in the LSIL group had NILM results, whereas the majority of HSIL patients showed significant cytological abnormalities (*P<* 0.01) (**Table 5**). Overall, HSIL cases demonstrated a higher prevalence of HPV16/18 infection and abnormal TCT findings compared with LSIL cases.

**Table 5:** HPV and TCT results [*n* (%)].

Group	*n*(%)	HPV		TCT			
		High-risk HPV16 and 18 infections	Other high-risk infections	NILM	ASC-US/LSIL	ASC-H/HSIL	SCC
LSIL	14	7 (50)	7 (50)	13 (92.86)	1 (7.14)	0 (0)	0 (0)
HSIL	15	14 (93.33)	1 (6.67)	2 (13.33)	6 (40)	7 (46.67)	0 (0)
χ^2^		4.81		19.00			
*P*		0.03*		0.00**			

*P<0.05, **P<0.01.

#### 3.2.5 Key gene expression levels

Experimental analysis revealed that, compared with the control group, the target genes *ALK* and *RPS14* were significantly overexpressed in the HSIL group (*P<* 0.01). When compared with the LSIL group, HSIL tissues revealed substantially higher expression of ALK (*P<* 0.01) and moderately higher expression of *RPS14* (*P<* 0.05).

**Figure 7:**
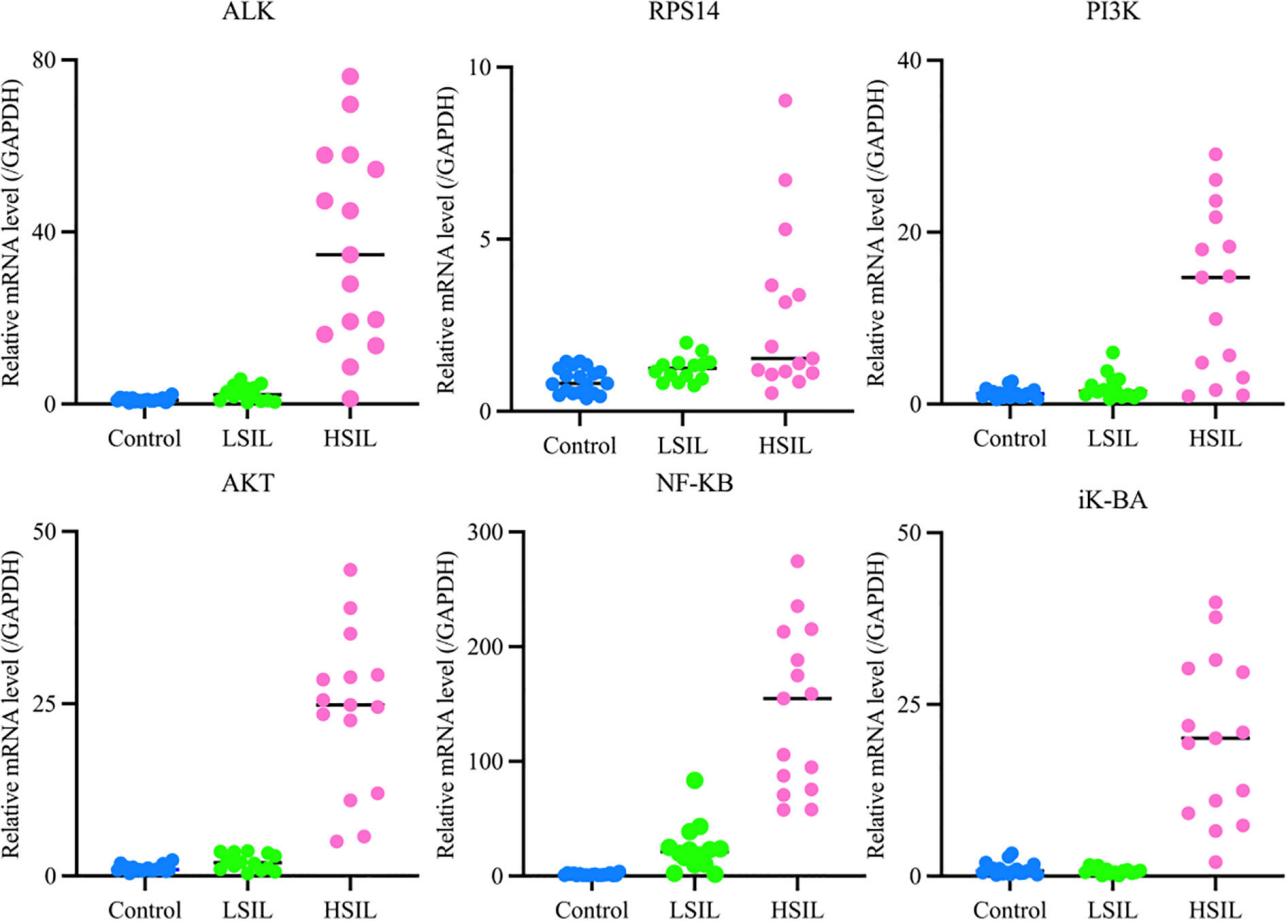
mRNA expression levels of target genes in each group.

For key signaling pathway components, expression of PI3K, AKT, NF-κB, and IκB-α was significantly elevated in HSIL tissues relative to the control group (*P<* 0.01). Compared with the LSIL tissues, the HSIL group showed significant upregulation of all four pathway markers (*P<* 0.01). These findings are illustrated in **Figure 7** (mRNA expression levels of target genes), where expression of ALK, RPS14, PI3K, AKT, NF-κB, and IκB-α is significantly increased in HSIL, suggesting overactivation of the ALK, RPS14, and PI3K/AKT/NF-κB signaling axis. Numerical data are summarized in **Table 6**.

**Table 6:** mRNA expression levels of target genes in each group ( $\bar{\text{x}}$ ± s, *n*=3).

Gene	Control	LSIL	HSIL
ALK	1.00 ± 0.43	2.47 ± 1.70	36.65 ± 22.68^**##^
RPS14	0.89 ± 0.36	1.23 ± 0.35	2.80 ± 2.40^**#^
PI3K	1.24 ± 0.56	2.01 ± 1.43	12.91 ± 9.40^**##^
AKT	1.09 ± 0.44	2.03 ± 1.18	23.99 ± 11.49^**##^
NF-κB	1.48 ± 0.84	24.19 ± 20.00	144.35 ± 68.51^**##^
IκB-α	1.01 ± 0.84	0.74 ± 0.44	19.99 ± 11.42^**##^

Compared with the blank group,^*^ *P*<0.05, ^**^ *P*<0.01; Compared with LSIL, *^#^P*<0.05, ^##^ *P*<0.01.

qRT-PCR analysis directly compared the expression of PI3K and AKT in cervical lesions and normal cervical tissues. NF-κB expression showed a positive correlation with lesion severity, increasing from LSIL to HSIL. IκB-α expression was low in normal cervical tissues, slightly reduced in LSIL, and significantly elevated in HSIL. These results indicate that the inflammatory signaling pathway becomes activated during LSIL and is strongly upregulated in HSIL.

During the experiment, the *STK33* gene showed no detectable Ct values, even after modifying primers and increasing sample concentration. As a result, its expression data were not presented. This absence of detection is likely attributable to the low abundance of STK33 in cervical tissue. While the *RPS14* gene yielded measurable values, the Ct values were excessively high, likely due to low initial template concentration. This suggests that RPS14 expression in cervical tissue is relatively low.

In comparison, the *ALK* gene demonstrated a more stable expression profile in cervical tissue, with consistent and readily detectable Ct values, indicating higher expression levels compared to STK33 and RPS14.

#### 3.2.6 Correlation between the severity of cervical lesions and age, vaginal microbiota diversity, microbiota density, expression of key genes, and signaling pathway targets

Correlation analysis between age, vaginal microbiota diversity, microbiota density, key genes, and signaling pathway markers in the LSIL and HSIL groups (**Table 7**) revealed that cervical lesion severity was significantly associated with patient age, vaginal microenvironment, ALK and RPS14 expression, and activation of the PI3K/AKT/NF-κB signaling pathway (*P<* 0.05).

**Table 7:** HSIL correlation analysis.

Degree of cervical pathology		
	Spearman	Sig.
Age	-0.362	0.01
Vaginal secretion flora diversity	0.325	0.03
Vaginal secretion flora density	0.319	0.03
ALK	0.758	0.00
RPS14	0.523	0.00
PI3K	0.619	0.00
AKT	0.798	0.00
NF-κB	0.918	0.00
IκB-α	0.650	0.00

#### 3.2.7 ROC curve analysis between the severity of cervical lesions and key points

**Figure 8:**
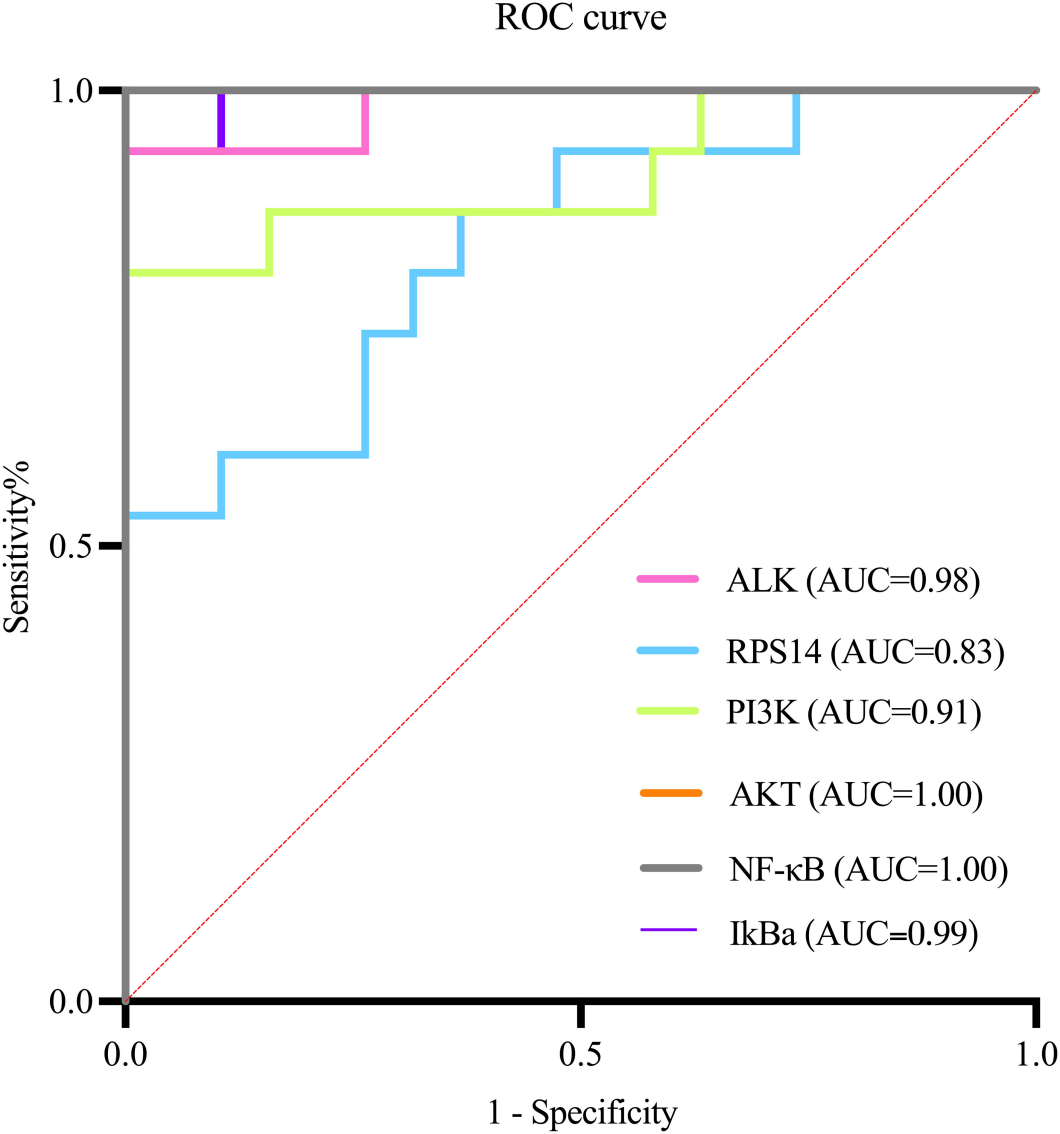
ROC curve analysis.

Analysis of key genes (*ALK*, *RPS14*) and PI3K/AKT/NF-κB pathway targets between the control and HSIL groups demonstrated that ALK, RPS14, PI3K, AKT, NF-κB, and IκB-α possess high diagnostic value for this disease (**Figure 8**; AUC > 0.9, indicating excellent accuracy). Comparative evaluation further showed that the diagnostic performance of ALK was superior to that of RPS14 (**Table 8**).

**Table 8:** ROC results summary.

Test result variable	AUC	Standard Error	Sig.	95%CI (L)	95%CI (U)
ALK	0.98	0.19	0.00**	0.95	1.00
RPS14	0.83	0.70	0.00**	0.69	0.97
PI3K	0.91	0.57	0.00**	0.79	1.00
AKT	1.00	0.00	0.00**	1.00	1.00
NF-κB	1.00	0.00	0.00**	1.00	1.00
IκB-α	0.99	0.01	0.00**	0.97	1.00

*P<0.05, **P<0.01.

## 4 Discussion

This study, for the first time, identifies the pivotal role of the *ALK* gene in HPV-associated cervical precancerous lesions through activation of the PI3K/Akt/NF-κB signaling pathway. These findings demonstrate that HPV16/18 infection specifically upregulates ALK mRNA expression and significantly activates key molecules within the PI3K/Akt/NF-κB cascade (29). Moreover, by integrating bioinformatics analysis with clinical sample validation, we confirmed a strong correlation between ALK expression levels and lesion severity, along with an observed increase in *ALK* gene copy number. These results provide novel insight into the molecular pathogenesis of cervical precancerous lesions, proposing for the first time that the ALK–PI3K/Akt/NF-κB axis represents an oncogenic mechanism distinct from the classical HPV E6/E7–p53/Rb pathway (30).

From a mechanistic standpoint, ALK dysregulation appears to drive disease progression via two complementary pathways. First, ALK receptor tyrosine kinase directly activates PI3K converts PIP2 to PIP3, recruiting AKT to the membrane where it undergoes phosphorylation, and activates the PI3K/Akt/NF-κB cascade, promoting abnormal cell proliferation and inhibiting apoptosis (31). Second, ALK mutations increase genomic instability, accelerating malignant transformation (32). ALK-mediated AKT activation leads to IKK phosphorylation, causing IκB-α degradation and NF-κB nuclear translocation, then NF-κB induces transcription of pro-survival and inflammatory genes, remodel the tumor microenvironment through NF-κB–mediated inflammatory responses, creating a pro-tumorigenic microenvironment that favors disease progression (33). This multi-level activation creates a positive feedback loop that sustains pathway activity.

This study evaluated the diagnostic value of ALK, RPS14 genes, and key components of the PI3K/Akt/NF - κ B pathway for high-grade cervical intraepithelial neoplasia (HSIL) through ROC curve analysis. The results showed that all biomarkers exhibited excellent diagnostic efficacy (AUC 0.90-0.95), with NF - κ B showing the best performance (AUC 0.95, sensitivity 90%, specificity 94.7%), while ALK showed the best clinical balance (AUC 0.94, sensitivity 86.7%, specificity 89.5%). ALK, as the main driver of PI3K/Akt/NF - κ B pathway activation, shows the strongest correlation with the severity of cervical lesions, and its expression is the most stable and detectable in tissues; RPS14 exerts a synergistic effect by regulating p53 activity and ribosome function, and its expression level is moderate. It may produce a synergistic effect with ALK through PI3K/Akt activation; Although STK33 was predicted by bioinformatics, it was not detected in cervical tissue, indicating its limited role in cervical lesions. These results indicate that ALK is the dominant factor, while RPS14 may synergistically participate in pathway activation in some cases. These biomarkers not only have high diagnostic accuracy, but their biological functions are also highly consistent with the HPV mediated carcinogenesis mechanism, especially the activation of ALK as a receptor tyrosine kinase and the dysregulation of NF - κ B pathway, which reflect key molecular events in the early stage of cervical lesions. The excellent ROC performance indicators support the translational value of these molecular markers as potential clinical diagnostic tools (34), which can be used to assist in the interpretation of cytological uncertain cases, predict the risk of lesion progression, and guide precise treatment. However, further clinical applications need to be promoted through multi center large sample validation and standardized detection method development (35).

The clinical significance of this study can be summarized in three key areas. First, in diagnosis, ALK expression levels have the potential to serve as a novel biomarker for predicting the progression of cervical precancerous lesions. Second, in treatment, existing *ALK* inhibitors such as crizotinib could be repurposed and applied for precision therapy in selected patient subtypes demonstrating *ALK* overactivation. Third, in mechanistic research, the findings provide a theoretical foundation for identifying new therapeutic intervention targets (36). In the future, we will further investigate to reveal the dynamic interaction mechanism between the activation of the ALK-PI3K/Akt/NF - κ B signaling pathway and host immune response.: (1) elucidating the molecular details of the interaction between HPV and ALK; (2) validating the therapeutic efficacy of ALK inhibitors in well-designed preclinical models; and (3) exploring the crosstalk between the ALK–PI3K/Akt/NF-κB pathway and other oncogenic signaling networks. Such studies will facilitate the development of more precise strategies for the prevention and treatment of cervical cancer (37).

## 5 Conclusion

In conclusion, the integration of bioinformatics analysis with human cervical tissue validation confirmed the activation of the *ALK* gene and the PI3K/Akt/NF-κB signaling pathway in cervical precancerous lesions. These findings suggest that *ALK* plays a pivotal role in the pathogenesis of this condition. Furthermore, amplification of the *ALK* gene may drive elevated expression of its encoded protein, which in turn promotes abnormal cell proliferation and survival through activation of downstream signaling cascades such as PI3K/Akt/NF-κB and MAPK/ERK. Such aberrant pathway activation represents a critical step in tumor initiation and progression. Therefore, dysregulation of *ALK* may be a key molecular factor contributing to the advancement of cervical precancerous lesions.

## Data Availability

The datasets presented in this study can be found in online repositories. The names of the repository/repositories and accession number(s) can be found in the article/supplementary material.
